# Cardiac involvement in hypereosinophilic syndromes detected by cardiac magnetic resonance imaging

**DOI:** 10.1186/1532-429X-17-S1-Q75

**Published:** 2015-02-03

**Authors:** Constanze Merten, Hans Beurich, Dirk Zachow, Fabian Arndt, Frank Moosig, Gert Richardt

**Affiliations:** 1 Cardiology, Herzzentrum Bad Segeberg, Bad Segeberg, Germany; 2 Radiology, Segeberger Kliniken, Bad Segeberg, Germany; 3 Rheumatology and Immunology, Klinikum Bad Bramstedt, Bad Bramstedt, Germany

## Background

The Hypereosinophilic Syndromes (HES) are a heterogeneous group of rare diseases characterized by blood hypereosinophilia and eosinophil-related organ damage with the Churg-Strauss syndrome as the vasculitic form of disease. Cardiac involvement was reported in 20-50% in earlier series. Yet, with improved diagnostic methods, the frequency of cardiac involvement is expected to be even higher. We therefore sought to evaluate the frequency and different forms of presentation of cardiac manifestations of HES using cardiac MRI.

## Methods

Forty-five patients diagnosed with HES and suspected to suffer from cardiac involvement based on clinical symptoms, echocardiography and laboratory findings were referred to our department for cardiac MRI. MRI analysis included standard cine sequences for the assessment of LV function, volumes and mass. T2-weighted imaging was conducted for the evaluation of myocardial oedema. Fat suppression sequences were used to differentiate between pericardial effusion (PE) and pericardial fatty tissue. Finally, gadolinium late enhancement (LE) imaging was conducted.

## Results

Of the 45 patients 15 were male (33%). Their mean age was 50.5±14 years. A subgroup of 20 patients also underwent coronary angiography. Relevant coronary artery disease was excluded in all but one patient who had a significant stenosis of a marginal branch.

**Figure 1:**
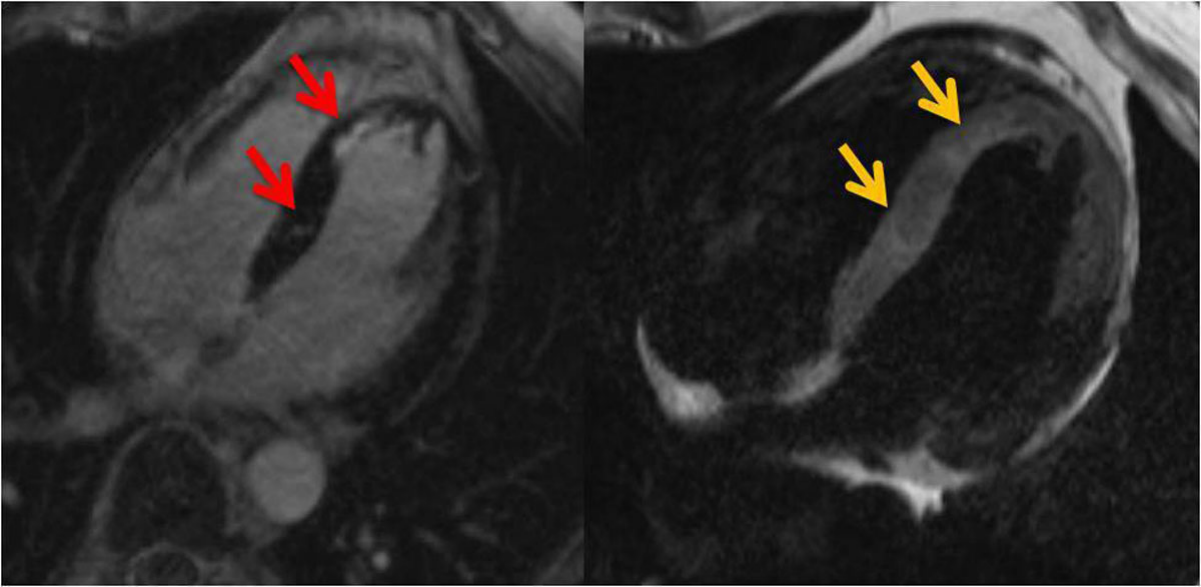
Late enhancement and corresponding myocardial oedema in a patient with cardiac involvement of HES.

**Figure 2:**
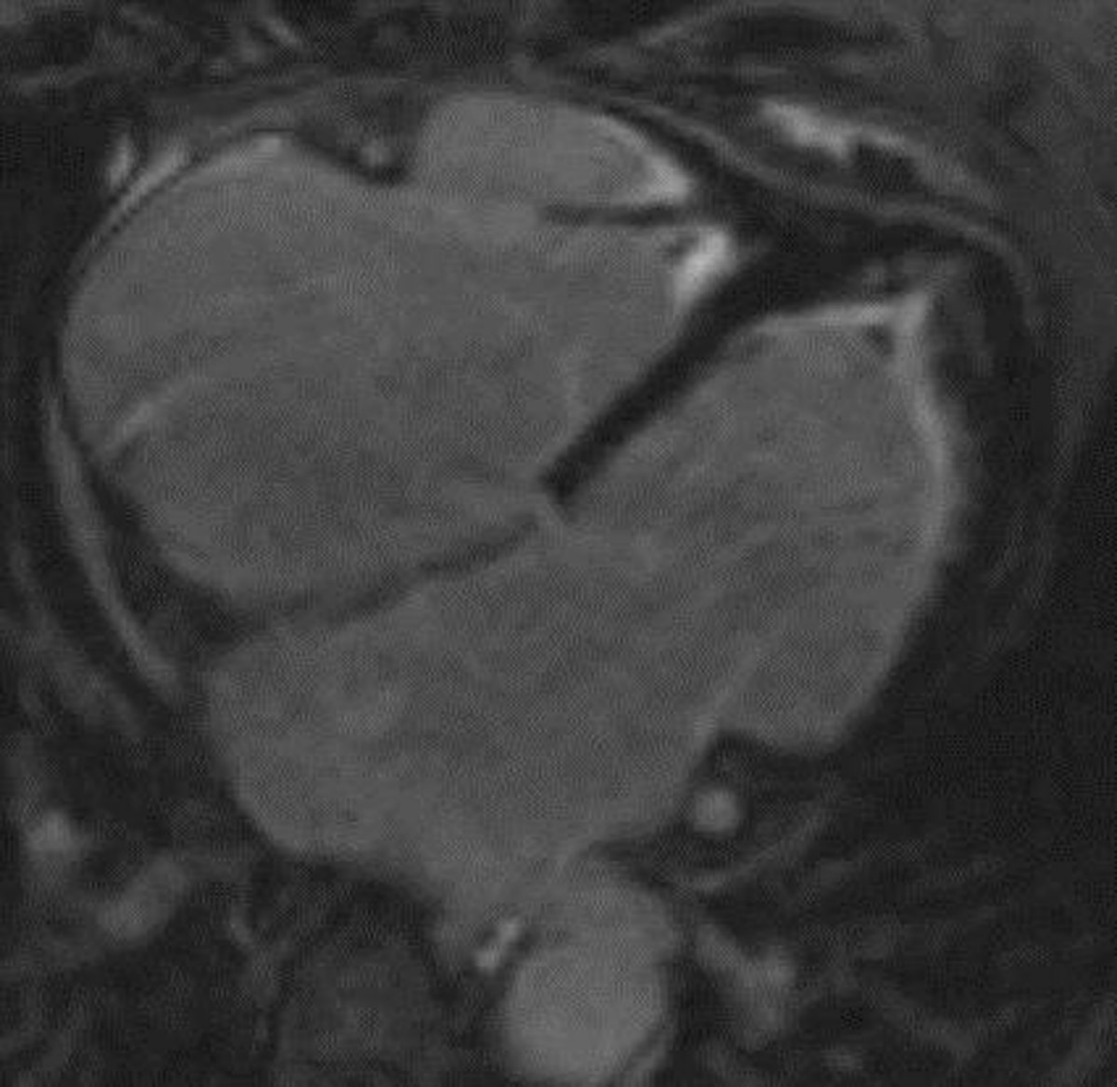
LE image of a patient with severe endomyocardial fibrosis.

Nine patients (20%) had a normal cardiac MRI scan with normal LV function and without any signs of myocardial oedema, PE or fibrosis. Three patients (7%) showed normal MRI with the exception of a small, non-circumferential PE of max. 5mm. In further 12 patients a PE ranging from 5 to 30mm was associated with other pathological findings. Analysis of LV function revealed a slightly impaired systolic LV function with a mean ejection fraction (EF) of 51.8±12.9%. EF was reduced (<55%) in 22 patients (49%). LE of the myocardium was observed in 30 patients (67%). All showed a non-ischemic- pattern of LE (fig. 1, red arrows) or multiple small endocardial LE zones not compatible with myocardial infarction due to coronary artery disease. Ten patients (22%) showed hyperintense areas in T2-weighted images indicative of myocardial oedema (fig. 1, yellow arrows). Finally, 4 patients (9%) showed the typical pattern of endomyocadial fibrosis with oedema and contrast enhancement of the myocardial / endocardial border zone and thrombotic material at the left and right ventricular apex (fig. 2).

## Conclusions

Cardiac MRI revealed abnormalities in 80% of patients with HES and suspected cardiac involvement. Yet, the pattern of cardiac manifestations varied significantly. More than half of the patients showed cardiomyopathy with a reduced systolic LV function. Non-ischemic-type LE was present in the majority of patients and was associated in one third of cases with myocardial oedema reflecting acute inflammation. The unique pattern of endomyocardial fibrosis involving oedema, fibrosis and sedimentation of thrombotic material was observed in 9% of the study cohort.

## Funding

None.

